# Treatment outcomes of applying external fixator on distal radius fractures: a randomized clinical trial to compare between two directions of force exertion in parallel to radius shaft and perpendicular to the distal radius articular surface

**DOI:** 10.1186/s12891-023-06358-1

**Published:** 2023-04-12

**Authors:** Davood Jafari, Ali Birjandinejad, Mahla Daliri, Kimia Emami, Ali Moradi

**Affiliations:** Orthopedics Research Center, Mashhad University of Medical Sciences, Mashhad, Iran

**Keywords:** Distal radius fracture, External fixator, Distraction force, Perpendicular, Clinical outcome, Radiological outcome

## Abstract

**Background:**

External fixation has been one of the conventional managements of unstable distal radius fracture. The main aim of this paper is to compare two methods of applying distractive force along the radius shaft versus perpendicular to the distal radius articular surface.

**Design:**

Sixty patients with unstable distal radius fracture were included in present clinical trial and randomized in two groups, using block randomization method. In group A (first arm), distraction force was exerted parallel to the radius shaft. In group B (second arm), the external fixator was adjusted based on radial and palmar tilt of the mean population healthy wrist so that distraction was exerted perpendicular to the wrist articular surface.

**Methods:**

Radiological and clinical parameters were evaluated in both groups of patients pre-operatively, immediately after surgery, and 6 weeks post-operatively. We also followed up patients clinically at 12 weeks after surgery. Patient-Rated Wrist Evaluation (PRWE), Mayo wrist score, and Quick Disabilities of the Arm, Shoulder and Hand (DASH) questionnaires were used in order to assess patients’ clinical and functional states.

**Results:**

The method used in group B resulted in better improvement of palmar tilt both immediately (*P* = 0.007) and at 6 weeks follow up (*P* = 0.013) post-operatively in comparison with patients in group A. Radius height and radial inclination were also better restored when using the proposed modified method (*P* = 0.001 and < 0.001, respectively). There was no difference in any of clinical results (range of motion, grip strength, PRWE, Mayo, and DASH scores) between two groups of study, 12 weeks after surgery.

**Conclusion:**

Applying distractive force perpendicular to the distal radius articular surface seems to improve some radiological outcomes, probably due to better reduction maintenance, when compared with the technique of applying distraction force along distal radius shaft axis.

**Level of Evidence:**

Level I (clinical trial study).

**Trial registration:**

This study is registered at Iranian Registry of Clinical Trials (IRCT) with approval code of IRCT20200313046759N1.

## Introduction

Distal radius fractures (DRFs) are among the most common fracture types in emergency setting [1, 2]. To treat DRF, there is a major shift from external to open fixation procedures [3], despite the absence of strong evidence in favor of improvement in clinical and functional outcomes with open fixation [4–6]. There is this meta-analysis found no significant difference in clinical outcomes between C-type DRF managed with locking plates and external fixators (EF) [7]. External fixation of distal radius fractures is advantageous in circumstances where soft tissue loss, wound contamination, and concomitant medical conditions make internal fixation procedures ineffective [8]. Several benefits of employing an EF have been described: anatomical reduction of the fracture under fluoroscopic vision; increase in reduction through ligamentotaxis, with the capacity to maintain the reduction until the fracture heals; simple hardware placement; minimal operating X-ray exposure; and reduced in surgical operation time [9]. Despite more favorable results from non-bridging EF (NBEF) in recent clinical trials, this technique has some limitations including efficacy in a select set of fractures, technical complexity (only enabling success in expert hands), the possibility of pin pullout from osteoporotic bone, and the possibility of extensor tendon damage [10, 11]. Bridging EF (BEF) is a traditional treatment, especially when the fracture is unstable [12–14]. However, this approach, which is based on the ligamentotaxis theory, is challenged for promoting palmar tilt loss and limiting wrist motion due to the cross-articular EF [15]. Another issue that affects treatment success rate due to joint stiffness is over-distraction [16–18]. This study specifically tries to address the issue with palmar tilt in BEFs application, which is a significant indicator of clinical prognosis in distal radius fractures [19].

**Fig. 1:**
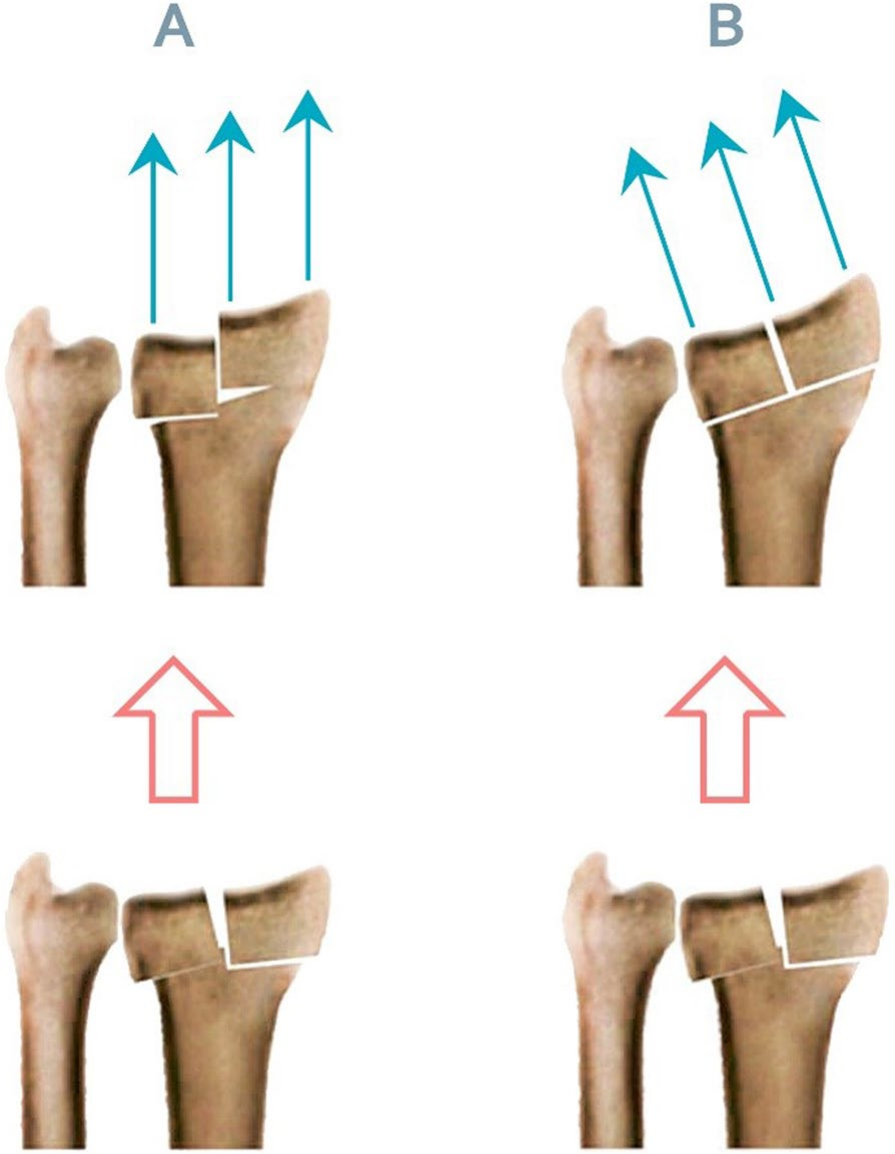
**A-B.** Illustration of the theoretical modification in external fixation directed force: force in parallel to radius shaft, which causes shear stress, and therefore articular step **A**, force perpendicular to the distal radius joint surface, which shows more proper reduction **B**

Palmar tilt restoration limit has been previously studied by Agee et al. who applied a multiplanar reduction strategy employing unilateral external fixation devices [20, 21]. We seek to determine the anatomic angles pre-operatively and apply the EF with set angles to avoid intra-operative trial and error, and hope to finally define an standard for the multiplanar BEF planes’ setup. We have hypothesized that applying distraction force perpendicular to the wrist joint, instead of parallel to radius shaft, improve the taxis and, therefore, BEF method related complications. Our prior case series study investigates this issue [22]. According to this hypothesis, when using the BEF, the indicated adjustment in the direction of the distractive force may avoid the shearing force exerted parallel to the articular surface and, as a result, displacement of the radial side fragments and articular step (Fig. 1). Thus, having the wrist joint in flexion and ulnar deviation during fixation, in accordance with palmar tilt and radial inclination prior to fracture, may improve the treatment outcomes [22]. Furthermore, with this adjusted force direction, we may potentially require less force application, reducing the over-distraction risk. Present study is a controlled clinical trial study to compare this method with conventional BEF technique, clinically and radiologically.

*Questions/purposes* This randomized clinical trial study set out to compare 1) radiological ( including palmar tilt, radial inclination, radius height, wrist alignment, and articular step) and 2) clinical outcomes (including grip strength, pain intensity, hand function, and wrist range of motion) between two techniques of applying longitudinal distraction force along distal radius axis as well as perpendicular to the wrist articular surface.

## Materials and methods

### Study setting

This randomised clinical trial was conducted in two tertiary hospitals. A random sample of patients with distal radius fracture were recruited between 2020 and 2021. They were provided with and signed written informed consent forms before enrolment. Ethical approval for this study was obtained from the related Ethics Committee (approval number: IR.MUMS.REC.1397.697). This study is registered at Iranian Registry of Clinical Trials (IRCT) with approval code of IRCT20200313046759N1 at 25/10/2021. The conduction of this research accords with the Declaration of Helsinki and adheres to the CONSORT guidelines [23]. Our unit’s most common standard care for unstable distal radius fractures is percutaneous pinning (PCP) and external fixation. The other option is internal fixation using plates. In our consent form we described both methods (internal and external Fixation) and their advantages and disadvantages, so patients could choose between them before entring the study. Patients who agreed with PCP and external fixation then enrolled in present study.

### Patients

**Fig. 2:**
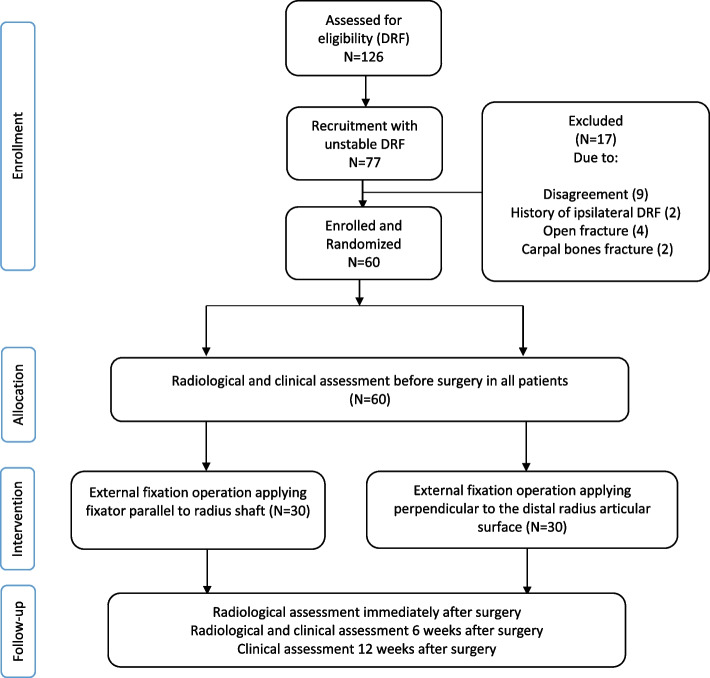
Flow chart presenting parallel randomised trial of two groups

In this parallel-designed randomised clinical trial, patients who were attending the hospital emergency department with acute unilateral distal radius fractures were randomly recruited by our hand surgeon (A. M). A priori sample size compute was conducted using G*power 3.1.9.4, with the effect size of 0.84. Based on a similar RCT study result for palmar tilt [24], alpha error of 0.05, power of 95%, and 1:1 ratio allocation; 38 patients in each group was calculated. 77 patients with unstable fracture were considered based on having one or more of the following criteria: intra-articular radiocarpal fracture, over 20° of dorsal angulation, dorsal comminution, and more than 5 mm shortening [25]. In case they had the indication of treatment with external fixator, they were contacted and informed about the study. Of those, 68 patients agreed to participate (agreed with PCP and external fixation surgery), and were provided with written informed consent forms. The exclusion criteria were patients with prior history of ipsilateral DRF, inflammatory diseases in affected wrist, open fracture, and concomitant carpal bones fracture. Accordingly, 8 patients were further excluded, leaving 60 patients as study sample for final evaluation (Fig. 2).

### Descriptive data

Finally, 60 patients were analysed (30 patients in each group) by original assigned groups. Demographic data including age, gender, and basic clinical data, such as the involved side and fracture type based on Fernandez classification [26] were gathered. The most frequent types were type 1 (32 patients) and type 3 (22 patients). Demographic data did not differ between two groups of patients (Table 1).

**Table 1:** Demographic and pre-operation radiological parameters data (*N* = 60)

Variable	Group A (*N* = 30)Mean (SD)	Group B (*N* = 30)Mean (SD)	*P* value
Age (Year) ^a^	44.93 (SD 14.49)	43.90 (SD 11.07)	0.757
Sex % (Male) ^b^	53.30%	56.70%	1.000
Involved side % (Right) ^b^	56.70%	53.30%	1.000
Fernandez classification fracture type (%) ^c^			
* Type 1*	23.30%	30.00%	0.154
* Type 2*	5.00%	0.00%	
* Type 3*	16.70%	20.00%	
* Type 4*	3.30%	0.00%	
* Type 5*	1.70%	0.00%	
VAS pain score ^d^	6.20 (SD 2.31)	6.37 (SD 1.73)	0.676
Radius height ^d^	2.60 (SD 1.81)	3.47 (SD 2.43)	0.113
Palmar tilt ^d^	18.23 (SD 8.05)	17.10 (SD 11.81)	0.237
Radial inclination ^a^	16.57 (SD 8.34)	16.30 (SD 5.15)	0.882
Wrist alignment ^a^	17.50 (SD 7.81)	16.80 (SD 7.04)	0.717
Articular step ^d^	0.43 (SD 0.73)	0.40 (SD 0.67)	0.934

Visual Analogue Score (VAS)

^a^Independent T test, ^b^Fischer’s exact test, ^c^Chi square test, ^d^Mann Whitney test

### Study design

Before operation, imaging study was performed for measuring the radiological parameters of joint displacement (palmar tilt, radial high, radial inclination, articular step and wrist alignment). These measurements were conducted before allocation of patients to either group in order to ensure blindness of data. The letters A and B were used for conventional treatment; the letters C and D were used considered for our proposed method of treatment, before randomization to conceal the group assignment. Patients were then randomly allocated (1:1 ratio) to one of the two groups using block randomization method (block size: 4, block number: 15, permutation number: 24) by a biostatistician, and one group was set to undergo external fixation operation applying fixator parallel to radius shaft (group A = 30) and the other group with external fixator perpendicular to the distal radius articular surface (group B = 30) (Fig. 2).

### Operation

**Fig. 3:**
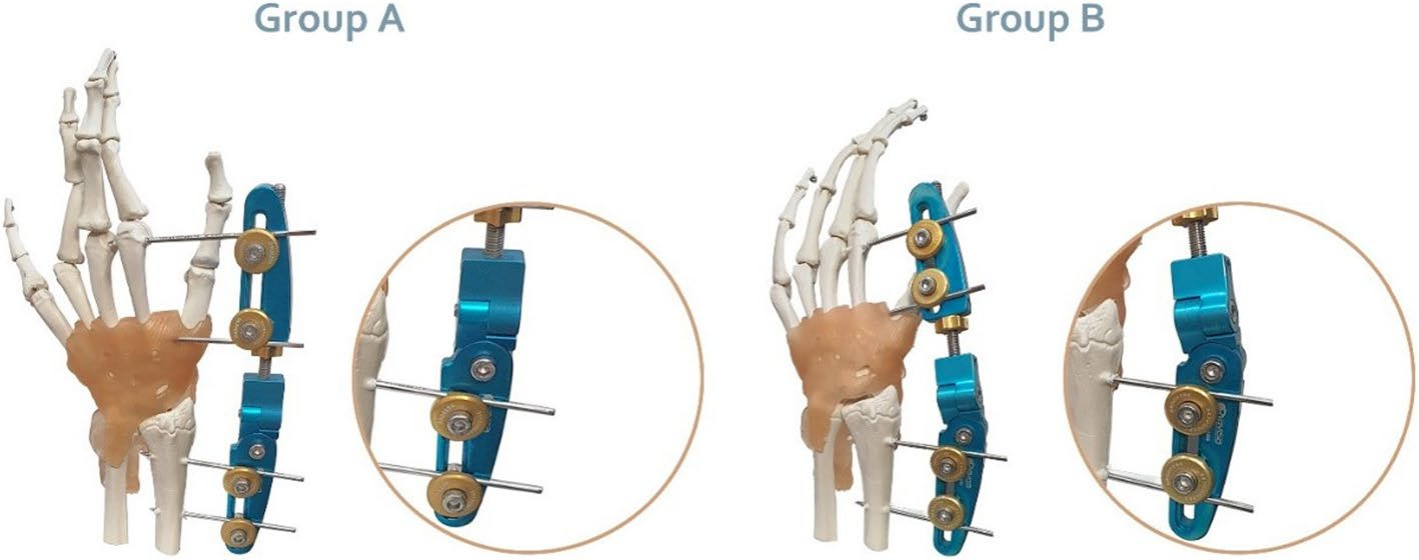
**A-B** The external fixator setting: for patients in group A on whom distraction force was applied parallel to radius shaft (**A**), for patients in group B on whom distraction force was applied perpendicular to the wrist joint (**B**)

All patients were hospitalized and were initially managed by long forearm splint. The operation procedure was performed with the patient in the supine position following induction of general anaesthesia. Then, the arm was prepped and draped. Using the appropriate manoeuvre of traction, flexion, and ulnar deviation, closed reduction was performed. Before fixation, re-imaging by fluoroscopy was done to ensure appropriate reduction. Then, with one radial side and one ulnar side pins, closed fixation was performed. Closed reduction and correct fixation (placement of the pins) were again verified with fluoroscopy and proximal schanz pins were inserted in radius shaft. First, we determined the entrance points for pins in lateral side, and then through a 5-mm skin incision and soft tissue dissection to the bone (using 11 bistoury). Two separated 2.5 mm schanz pins were placed perpendicular to the radius shaft and proximal to the fracture line. Via a limited incision on second metacarpal bone base, a 2.5 mm schanz pin was placed in metaphysis-diaphysis junction and extended through third metacarpal lateral cortex. Another 2.5 mm schanz pin was placed in second metacarpal shaft. For fixation and distraction, we used a pre-fabricated external fixator with two adjustable joints for palmar tilt and radial deviation. External fixator was mounted to the schanz pin using the two techniques below:

1. All external fixator indices were set on zero and, longitudinal distraction force was applied parallel to distal radius axis (group A) (Fig. 3-A).
2. The external fixator indices were set in accordance with mean normal population wrist radial inclination (24 degrees) and palmar tilt (10 degrees). Then, distraction force was applied perpendicular to the wrist joint (group B) (Fig. 3-B). In order to make sure the distraction force is exerted perpendicular to the articular surface, we estimated the direction of force based on Mashhad population normal distal radius indices previously determined in Vaezi et al. [27] Accordingly, normal radial inclination and palmar tilt were considered 24 and 10 degrees, respectively.

After external fixator insertion, over distraction under guide of fluoroscopy was performed in a controlled way until 2 mm distraction occurred in radio-lunate joint [28]. We then gradually decreased the distraction to 1 mm.

### Post-operation management

To gather data, we further recorded radiological data parameters at 6 weeks follow up. The fixator was also removed at 6 weeks, after we clinically ensured that the union achieved. Patients’ clinical data were gathered at 6 and 12 weeks post-operatively. Follow-up data was gathered by a medical intern who is trained in orthopaedic research fields (KE). To ensure blindness, we asked her to complete and record measurements after EF extraction.

### Study outcomes

#### Primary outcome

The primary purpose of this research was to compare DRF-related radiological findings, more specifically palmar tilt, between two groups. To answer this, we took true posteroanterior (PA) and lateral distal radius radiographs, and recorded the indices of radial inclination and palmar tilt values, as well as joint displacement parameters (radial height, radial inclination, articular step, and wrist alignment) using IC Measure software (version 2.0.0.286, the imaging source, Bremen, Germany).

#### Secondary outcome

We were also looking to compare clinical outcomes in terms of hand strength, function, range of motion, and pain severity between two groups. To do this, patients’ clinical data using grip strength dynamometry and wrist range of motion, as well as validated questionnaires’ score of Quick Disabilities of the Arm, Shoulder and Hand (DASH), Mayo wrist score, Patient-Rated Wrist Evaluation (PRWE) and Pain Visual Analogue Score (VAS) were gathered at 6 and 12 weeks post-operatively.

### Tools (data sources)

**Fig. 4:**
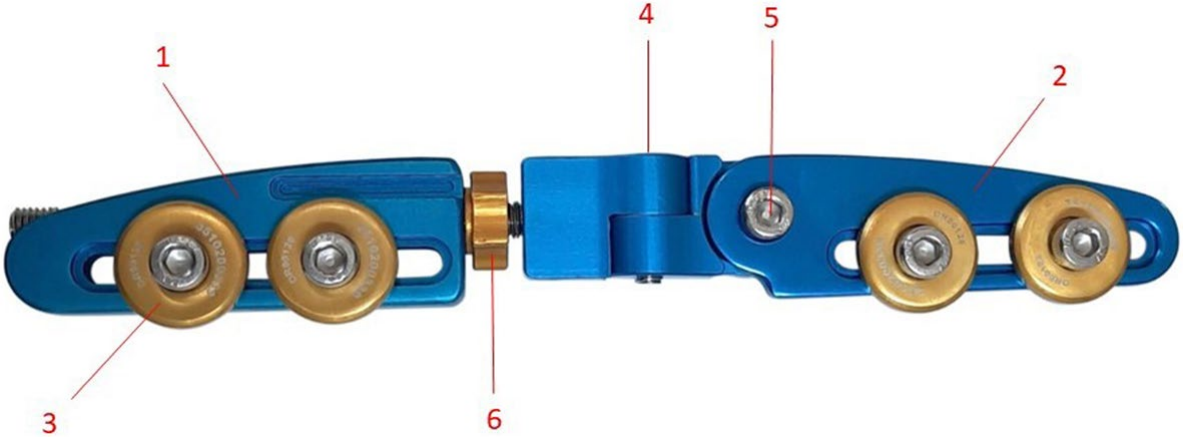
The biplanar adjustable joint bridging external fixator (Avisa Co., Mashhad, Iran) which consists of: 1- Metacarpal plate, 2- Radius plate, 3- Clamp, 4-Distal joint (for palmar tilt), 5- Proximal joint (for radial inclination), 6- Distraction nut

We used Avisa biplanar adjustable joint bridging external fixator (Avisamedical, Mashhad, Iran, http://avisamedical.com/index.php/shop/external-fixators/dynamic-distal-radius-external-fixator). It consists of a radius fixing plate with two clamps, a metacarpus fixing plate with two clamps, and a coupling treaded bar. The radius plate includes two joints. The proximal joint has one degree of freedom along coronal plan (for radial deviation adjustment) and the distal joint has one degree of freedom on coronal plan (for palmar tilt adjustment). A single nut between two plates distracts the clamps (Fig. 4).

**Fig. 5:**
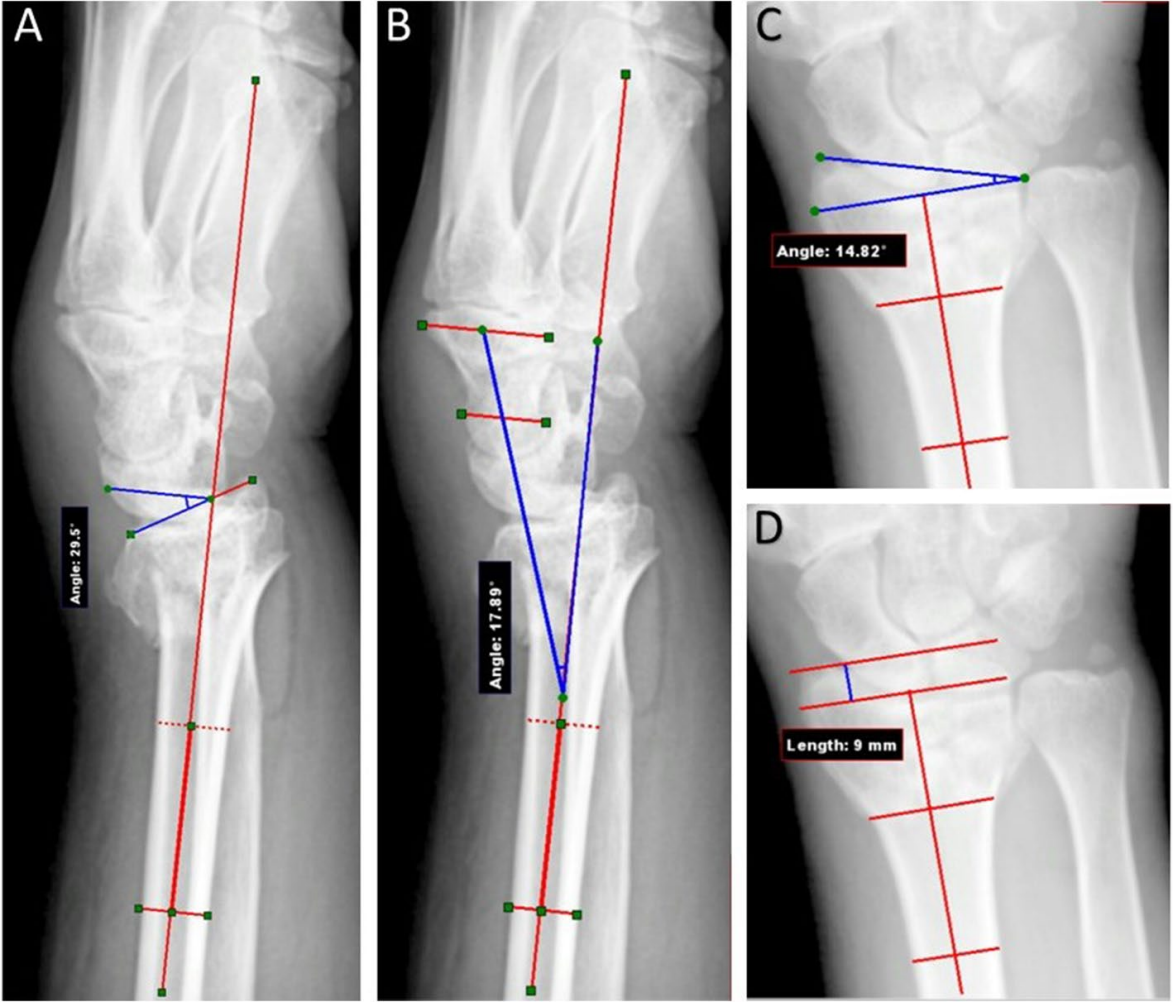
**A-D** Measurement of radiological parameters before the operation: palmar tilt (**A**), wrist alignment (**B**), radial inclination (**C**) and radius height (**D**)

Below are tools for measuring clinical variables. These data were recorded at 6 and then 12 weeks post-operatively:

- Goniometry: Wrist range of motion in six positions of flexion, extension, pronation, supination, radial deviation and ulnar deviation were measured three times, the mean of which was recorded.
- Grip strength: With the patients in sitting position, elbow in 90 degrees flexion and neutral forearm and wrist position, grip strength was measured three times with Lafayette hand evaluation kit, hydraulic grip dynamometer, Model J00109 (Lafayette, Sagamore Pkwy N Lafayette, IN 47,904, USA), the maximum of which was recorded.
- Patient-Rated Wrist Evaluation (PRWE) questionnaire: This questionnaire evaluates three factors of wrist pain, disability in activities of daily living, and disability with doing specific activities. It consists of 15 items and each item has ten scores; based on item scores, scale scores are calculated ranging from 0 (no pain or disability) to 100 (most severe pain and disability) [29]. We used the translated and validated version of the questionnaire in Persian [30].
- Quick Disabilities of the Arm, Shoulder and Hand (DASH) questionnaire: The Quick DASH questionnaire includes 11 items from the original 30-item DASH, evaluating upper limb symptoms and disabilities. Questions are about the patient’s ability to perform different activities, sleep quality, social and regular daily activities, pain severity, and tingling. Each item has five response options; based on item scores, scale scores are calculated ranging from 0 (no disability) to 100 (most severe disability) [31]. We used the translated and validated version of the questionnaire for Persian speakers [32].
- Mayo wrist score questionnaire: It assesses four domains of pain, satisfaction, wrist range of motion and grip strength. Each domain is scored from 0 to 25 points to produce a total score out of 100 points. Higher scores mean better function: Scores of 90–100 are interpreted as “excellent” function, 80–89 as “good”, 65–79 as “intermediate” and a score of less than 65 is considered “poor” [33]. The physician completed this questionnaire for patients, and thus the translated version was not required.
- Pain Visual Analogue Score (VAS): In order to quantify the pain severity, we used the VAS scale, scaled continuously from 0 (no pain) to 10 (worst pain) on a 10 cm scale [34, 35]. We then measured the distance from 0 to the point where patients marked their pain level in centimeters (cm).
- Radiography: Radiological parameters were measured three times by taking PA and lateral distal radius radiographs once before surgery and then immediately and 6 weeks post-operatively. Radiographs were performed under supervision of one of our researchers (KM) to reassure the radiology beam is perpendicular to sagittal plane of radius shaft. Radiological parameters were measured by a radiologist’s technician, who was blind to group allocation of patients. The measured parameters are:
- Radius palmar tilt: Taking lateral view, this parameter is the angle made by the line vertical to radius shaft and the line tangent to the volar to dorsal aspect of the distal radius (Fig. 5-A).
- Wrist malalignment: The angle between the lines drawn along the long axes of Capitate and radius from a lateral view (Fig. 5-B).
- Radial inclination: The angle between the line vertical to the radius shaft axis and the line that connects the distal radio-ulnar joint (the midpoint of volar and dorsal lips) with styloid process in PA view (Fig. 5-C).
- Radius height: Taking PA view, this parameter is the distance in millimeter between two parallel lines which are vertical to radius shaft. One line is drawn from level of the ulnar aspect of the articular surface (the midpoint of volar and dorsal lips) and the other from apex of radius styloid [Fig. 5-D]**.**
- Articular step: Measurement of depression or protuberance in joint surface using AP view. In fractures with multiple articular steps, we calculated this value by considering the most depressed and bulged steps among them.

### Statistical analysis

Data were analysed using SPSS (version 22). Quantitative data are reported as the mean ± SD, and qualitative data as number in percentage. After determination of variable data distribution with Kolmogrov-Smirnov test, comparison between two groups were performed using independent T-test when the data were normally distributed; otherwise the Mann Whitney test was used. In order to compare the data before and after surgery within each group, paired T-test was performed for variables which had normal distribution and Wilcoxon test for variables with non-normal distribution. P values less than 0.05 were considered statistically significant.

## Results

### Pre-operation

There was no difference in pain VAS score and radiological parameters between two study groups before surgery (Table 1).

### Post-operation

Palmar tilt was significantly different between two groups immediately after surgery. No other radiological parameters were notably different (Table 2).

**Table 2:** Comparison of radiological parameters between two methods immediately after surgery

Variable	Two groups (Mean (SD))		*P* value
	Group A	Group B	
Radius height ^a^	9.03 (SD 2.17)	9.40 (SD 2.18)	0.309
Palmar tilt ^a^	6.60 (SD 3.92)	8.93 (SD 4.27)	**0.007**
Radial inclination ^b^	23.33 (SD 4.10)	24.20 (SD 3.22)	0.366
Wrist alignment ^a^	11.57 (SD 3.91)	13.13 (SD 4.97)	0.327

^a^Mann Whitney test, ^b^Independent T test

### Six weeks post-operatively

There was a significant difference in palmar tilt (*P* = 0.013), radius height (*P* = 0.001) and radial inclination (*P* < 0.001) between group A and group B, 6 weeks after surgery. Other radiological and clinical (range of motion, grip strength and clinical questionnaire scores) parameters were not different (Table 3). Most patients were classified as poor (38 patients) and intermediate (14 patients) according to Mayo wrist score, and there was also no difference in this score between two groups.

**Table 3:** Comparison of radiological and clinical parameters between two methods 6 weeks after surgery

Parameters	Variable	Two groups (Mean (SD))		*P* value
		Group A	Group B	
Radiological parameters	Radius height ^b^	8.23 (SD 2.46)	10.47 (SD 2.62)	**0.001**
	Palmar tilt ^a^	7.03 (SD 3.57)	8.80 (SD 3.56)	**0.013**
	Radial inclination ^a^	20.10 (SD 3.29)	23.30 (SD 2.87)	** < 0.001**
	wrist alignment ^a^	12.00 (SD 3.97)	13.27 (SD 4.70)	0.311
	Articular step ^a^	0.10 (SD 0.31)	0.10 (SD 0.31)	1.000
Wrist range of motion	Flexion ^b^	48.17 (SD 16.32)	50.50 (SD 14.58)	0.562
	Extension ^b^	41.33 (SD 16.08)	39.33 (SD 15.85)	0.629
	Radial deviation ^a^	21.33 (SD 6.94)	21.83 (SD 6.63)	0.745
	Ulnar deviation ^a^	38.17 (SD 12.70)	39.50 (SD 11.92)	0.616
	Pronation ^a^	62.00 (SD 12.77)	61.50 (SD 15.15)	0.726
	Supination ^a^	66.50 (SD 14.63)	62.30 (SD 14.90)	0.239
Clinical scores	Grip strength ^b^	24.97 (SD 12.66)	25.73 (SD 12.37)	0.813
	DASH ^a^	31.18 (SD 21.60)	27.28 (SD 15.69)	0.711
	PRWE ^b^	38.93 (SD 18.29)	36.43 (SD 15.69)	0.572
	Mayo ^b^	56.67 (SD 20.36)	58.17 (SD 16.63)	0.756
	VAS ^a^	3.70 (SD 2.53)	3.40 (SD 1.17)	0.725

Quick Disabilities of the Arm, Shoulder and Hand (DASH), Patient−Rated Wrist Evaluation (PRWE) and Pain Visual Analogue Score (VAS)

^a^Mann Whitney test, ^b^Independent T test

### 12 weeks post-operatively

There was no difference in any of clinical parameters (range of motion, grip strength and clinical questionnaire scores) between two groups of study, 12 weeks after surgery (Table 4). At week 12, according to Mayo classification scores, patients with “good” function increased to 10 patients and with “excellent” function increased to 8 patients, but there was still no notable difference in this score between two groups. In terms of complications assessed at 12 weeks post-operatively, there were no notable difference between frequencies of any early complications (Table 5). Pin track infection was the most frequent complication (5 and 4 patients in group A and B, respectively). Two patients in group A and one patient in group B had simultaneous complications of pin track infection and pin loosening. Reflex sympathetic dystrophy and median neuropathy also co-existed in one patient in each group.

**Table 4:** Comparison of clinical parameters between two methods 12 weeks after surgery

Parameters	Variable	Two groups (Mean (SD))		*P* value
		Group A	Group B	
Wrist range of motion	Flexion ^b^	51.17 (SD 14.00)	54.33 (SD 11.87)	0.349
	Extension ^b^	45.83 (SD 13.07)	44.50 (SD 14.70)	0.712
	Radial deviation ^a^	22.83 (SD 6.25)	23.83 (SD 5.83)	0.594
	Ulnar deviation ^a^	38.30 (SD 13.48)	40.50 (SD 11.62)	0.425
	Pronation ^b^	64.33 (SD 10.96)	64.00 (SD 13.03)	0.915
	Supination ^a^	67.67 (SD 13.82)	65.83 (SD 12.87)	0.545
Clinical scores	Grip strength ^b^	27.70 (SD 12.40)	29.93 (SD 12.72)	0.494
	DASH ^a^	13.42 (SD 11.33)	11.28 (SD 8.79)	0.629
	PRWE ^a^	16.90 (SD 13.65)	14.58 (SD 11.31)	0.528
	Mayo ^a^	73.50 (SD 15.66)	72.50 (SD 12.23)	0.976
	VAS ^a^	1.13 (SD 1.43)	0.90 (SD 1.18)	0.625

Quick Disabilities of the Arm, Shoulder and Hand (DASH), Patient−Rated Wrist Evaluation (PRWE) and Pain Visual Analogue Score (VAS)

^a^Mann Whitney test, ^b^Independent T test

**Table 5:** Comparison of complications at 12 weeks post-operatively

Complication	Group A (*N* = 30)	Group B (*N* = 30)	*P* value
Pin track infection	5	4	0.718
Pin loosening	1	2	0.554
Transient median neuropathy	2	1	0.554
Radial neuropathy	1	0	0.313
Reflex sympathetic dystrophy	1	1	1.000

All analyses were performed using Chi square test

## Discussion

The challenges of external fixation in distal radius fractures include wrist and finger stiffness and reflex sympathetic dystrophy as the results of over distraction, malunion, acute carpal tunnel syndrome (CTS) and radius shortening [17, 18, 36, 37]. Research on the subject has been mostly restricted to limited comparisons of bridging with non-bridging fixators [37] or external versus internal fixation [14]. However, there is a paucity of literature on modifying EF force direction to optimize reduction and stability. The concept with our proposed method is to individualize anatomic reduction of the fracture fragments with exertion of force based on the patients’ contralateral healthy wrist indices. The results of present study, comparing direction of distraction force in parallel to the radius shaft with perpendicular to distal radius articular surface, indicated that palmar tilt, radial height and radial inclination were better reestablished when distraction is applied perpendicular to the joint surface. However, we could not find any difference in terms of clinical outcomes.

### Limitation

The study is limited by a relatively short follow up time. One probable weakness in the study methodology was the lack of uninjured hand radiographies to enable us to exert the distraction force exactly perpendicular to the injured hand articular surface based on each patient’s normal articular indices. This issue could not be addressed, because of the ethical limitation in terms of radiation dose. Thus, we considered the previously studied mean indices of the city population in Vaezi et al. [27] article to estimate the articular angles, and therefore apply the distraction force approximately perpendicular to the joint surface. Thirdly, the study did not evaluate the distraction force range when applying two methods. As described in methodology, we performed percutaneous pinning (PCP) prior to external fixation as the standard procedure for all patients. This may have obscured the radiological difference in articular space fragments to some extent. However, we hypothesize that if ethical principles would allow us to perform PCP after external fixation, we could note the difference in articular anatomy more precisely.

## Discussion

The DRF treatment goal is to re-establish wrist functionality and range of motion while preventing subsequent complication like osteoarthritis [38]. Previous research has demonstrated that this is accomplished by accurately reducing normal radial inclination, radial height, and volar tilt, and as a result, radial articular step with reduction of the distal radioulnar joint in intra-articular fractures [38, 39]. When compared with lockig plate, the radiological examination revealed that the BEF group had a poorer radial inclination [7].Comparing dynamic EF with static bridging or non-bridging EFs, no notable anatomic or clinical difference was found [40, 41]. However, the concept with developing and applying dynamic EF was early mobilization in prior research, rather than optimizing the force direction. According to traditional view, longitudinal distraction restores radial height and inclination, but not volar tilt. Only after sectioning the volar radioscaphocapitate and long radiolunate ligaments was palmar tilt restored in one investigation [42]. There is evidence in support of using external fixators that are adjustable in multiple planes for reestablishing the anatomic alignment and, therefore, maintenance of fracture reduction during healing [8, 20, 21]. The capitate's palmar translocation intrudes on the lunate's volar lip. The subsequent palmar rotation of the lunate promotes radius reduction via the radiocarpal ligament. Utilizing volar translocation of the wrist relative to the radius shaft axis, the use of a dynamic external fixation device reduces tension on the volar ligaments [43, 44]. Volar translational maneuver during application of longitudinal traction is said to help restoring baseline palmar tilt, and consequently avoiding finger stiffness and carpal tunnel syndrome [8, 45]. In a recent study, ligamentotaxis with external fixation resulted in moderate outcome in 28 percent and poor in 9 percent of the patients evaluated by Modified of Gartland and Werley Demerit point system [46]. On cadaveric distal radius fractures, a multiplanar reduction approach using unilateral external fixation methods could yield an adequate reduction [8]. During our pilot study in 2014, distraction force was applied perpendicular to the distal radius articular surface using BEF (having injured joint in palmar tilt and radial inclination, equal to the uninjured side, during fixation). We reported that outcomes of this method were comparable to studies of non-bridging EF or other combined methods in terms of clinical and functional parameters. We finally hypothesized that perhaps less needed traction to maintain the reduction and direction of the distraction force perpendicular to the articular surface led to restoration of articular compatibility [22]. One limitation of our prior pilot study was lack of a cohort group treated with conventional method of external fixation to compare outcomes, and this made us curious to conduct the present study. As the difference in radiological outcomes were more significant at 6 weeks follow up in present study, we can say that the proposed method was more successful in reduction collapse prevention, rather than in reduction creation at first place.

To sum up, our proposed method could restore palmar tilt, radial inclination and radius height more properly when compared with the prior external fixation technique. Since radiological difference in radius height and radial inclination was observed at 6 weeks follow up and not immediately post-operation, group B method was more successful in prevention of reduction collapse. It is necessary to mention that although the radiological parameters were statistically significant, but we are in doubt whether these amounts are also clinically significant. Maybe with longer follow ups, it will become evident that some delayed complications like late arthrosis, which causes pain and decreased range of motion, also subside.

## Conclusion

The results of this clinical trial suggest that using distraction force perpendicular to the distal radius articular surface during DRF fixation and union improves post-operative radiological indices’ maintenance, as compared to conventional approach. This is the first clinical report comparing two directions of distractive force, and it recommended that more research on this external fixation approach be conducted with longer patient follow-ups to determine whether clinical parameters improve or not.

## Data Availability

Correspondence and requests for materials should be addressed to Moradial@mums.ac.ir.
